# A novel system for introducing precisely-controlled, unanticipated gait perturbations for the study of stumble recovery

**DOI:** 10.1186/s12984-019-0527-7

**Published:** 2019-06-10

**Authors:** Shane T. King, Maura E. Eveld, Andrés Martínez, Karl E. Zelik, Michael Goldfarb

**Affiliations:** 1 Department of Mechanical Engineering, Vanderbilt University, Nashville, TN U.S.; 2 Department of Biomedical Engineering, Vanderbilt University, Nashville, TN U.S.; 3 Department of Physical Medicine & Rehabilitation, Vanderbilt University, Nashville, TN U.S.; 4 Department of Electrical Engineering, Vanderbilt University, Nashville, TN U.S.

**Keywords:** Falling, Trip, Stumble Apparatus, Joint Kinematics, Joint Kinetics

## Abstract

**Background:**

The experimental study of stumble recovery is essential to better understanding the reflexive mechanisms that help prevent falls as well as the deficiencies in fall-prone populations. This study would benefit from a system that can introduce perturbations that: 1) are realistic (e.g., obstacle disrupting the foot in swing phase), 2) are unanticipated by subjects, 3) are controllable in their timing, and 4) allow for kinematic and kinetic evaluation.

**Methods:**

A stumble perturbation system was designed that consists of an obstacle delivery apparatus that releases an obstacle onto a force-instrumented treadmill and a predictive targeting algorithm which controls the timing of the perturbation to the foot during swing phase. Seven healthy subjects were recruited to take part in an experimental protocol for system validation, which consisted of two sub-experiments. First, a perception experiment determined whether subjects could perceive the obstacle as it slid onto the treadmill belt. Second, a perturbation experiment assessed the timing accuracy of perturbations relative to a target percent swing input by the experimenter. Data from this experiment were then used to demonstrate that joint kinematics and kinetics could be computed before and after the perturbation.

**Results:**

Out of 168 perception trials (24 per subject), not a single obstacle was perceived entering the treadmill by the subjects. Out of 196 perturbation trials, 190 trials successfully induced a stumble event, with a mean targeting accuracy, relative to the desired percent swing, of 25 ms (6.2% of swing phase). Joint kinematic and kinetic results were then computed for three common stumble recovery strategies and shown to be qualitatively consistent with results from prior stumble studies conducted overground.

**Conclusions:**

The stumble perturbation system successfully introduced realistic obstacle perturbations that were unanticipated by subjects. The targeting accuracy substantially reduced mistrials (i.e., trials that did not elicit a stumble) compared to previous studies. This accuracy enables stumble recovery to be studied more systematically as a function of when the perturbation occurs during swing phase. Lastly, joint kinematic and kinetic estimates allow for a comprehensive analysis of stumble recovery biomechanics.

**Electronic supplementary material:**

The online version of this article (10.1186/s12984-019-0527-7) contains supplementary material, which is available to authorized users.

## Background

Falls due to tripping are a common cause of injury [1, 2]. The experimental study of tripping (i.e., stumbling) and the subsequent recovery response in healthy and impaired people is essential to better understanding the reflexive mechanisms that help prevent falls and the deficiencies in some populations that increase the proclivity for falls. Such studies are an important element of developing interventions that can reduce the likelihood or severity of falls, particularly in impaired populations.

An effective experimental system for studying stumble recovery would ideally have several characteristics. First, it should be able to introduce a perturbation to elicit a stumble. This perturbation should be delivered in a realistic manner. As such, the perturbation should be applied to the foot during swing phase using a three-dimensional object which must be cleared by the swing foot in order to recover, analogous to stumble events in daily life (e.g., stumbling on an uneven sidewalk or a toy on the floor). A perturbation of this nature is hereafter referred to as an obstacle perturbation. Second, the system should be capable of introducing repeated, unanticipated perturbations so that multiple responses can be characterized as a function of the experimental conditions and analyzed statistically. The repeated presentation of perturbations, however, is challenging because eliciting an authentic stumble response (i.e., a reflexive response) relies on the subject not anticipating each perturbation (i.e., the subject receiving no sensory cues indicating when or where the perturbation will occur). Third, the timing of the perturbation should be controllable, such that it can be targeted within a specific window of the gait cycle (i.e., to perturb the foot at different portions of swing phase). Controllable timing of the perturbation minimizes the number of mistrials in an experiment and allows for a more thorough analysis of stumble recovery responses. Fourth, the experimental system should enable measurement of kinematics and kinetics on both the perturbed (ipsilateral) and contralateral limbs before, during, and after the stumble in order to effectively assess recovery biomechanics.

Several systems have been developed to produce stumbles in human gait, including overground floor-deployable obstacle perturbations (e.g. [3–16]), overground rope-blocking (e.g. [17]), treadmill-based belt-deployable obstacle perturbations (e.g., [18–21]), treadmill-based rope-blocking (e.g. [22–26]), and treadmill-based belt-speed perturbations (e.g. [27–31]). Although these systems can each be effective in causing a person to stumble, each also entails some limitations with respect to the aforementioned desired characteristics of a system for studying stumble recovery. For instance, overground floor-deployable obstacle perturbations are an effective means of emulating the described conditions for obstacle perturbations, but are limited by constrained walkway length. In this type of setup, subjects are frequently aware of the nominal location of the perturbation, and as such introducing a large number of perturbations in an unexpected manner has been challenging. As described in [7], to avoid the possibility of subject anticipation, 79 subjects were recruited, each to be stumbled once; however, only 61 subjects were successfully stumbled, resulting in unusable data from 18 subjects. Further, it can be difficult to ensure a large number of strides between the initiation of walking and the introduction of the perturbation due to limited walkway lengths. Finally, in previous studies, the relative phasing between the subject and the obstacle has been determined by the subject at the start of the walkway, and once established, the timing of the perturbation has been difficult to control. Overground rope-blocking perturbations entail similar limitations due to walkway length, do not emulate a physical obstacle which must be cleared, and may complicate kinetic analysis due to the rope applying a force to the foot before and after the perturbation [17].

Other researchers have employed treadmill-based systems which largely address the challenges of limited walkway length associated with overground setups. Treadmill-based setups enable a large number of perturbations in a given session, provide an assurance of steady-state walking prior to the perturbation, and allow for better control of perturbation timing with respect to swing percentage. However, neither rope-blocking nor belt-speed perturbations recreate an obstacle perturbation due to the lack of a physical object to clear. Also, in the case of belt-speed perturbations, the perturbation is not applied to the swing foot, introducing both mechanical and sensory differences relative to an obstacle perturbation. The belt-deployable obstacle approach, as employed in [18–21], most closely resembles the ideal system characteristics detailed above. This previous system used an electromagnet to release an obstacle from a small height above the treadmill belt. However, a subject was easily able to detect when an obstacle was released onto the belt through the vibration produced by the impact, which introduced the potential for anticipatory responses. In [19, 20], the authors describe circumventing this issue by using a metal rod to strike the treadmill at irregular intervals, in order to disorient the subject as to which impact preceded the impending perturbation. This tactic may decrease the likelihood of an anticipatory response from the subject, but may not completely eliminate it. Further, the repeated impacts confound the calculation of kinetics prior to the perturbation.

It should be noted that of all the stumble studies previously described, only two have presented joint-level kinetic data. Ipsilateral limb kinetics were reported in [4], and contralateral limb kinetics were reported in [12]. The lack of reported joint-level kinetic data is likely due to the challenge of reliably measuring force and moment data in several of the aforementioned systems associated with stumble experimentation.

In this paper, the authors describe the design and methodology of a novel stumble perturbation system that 1) provides an obstacle perturbation (i.e., one similar to stumble events encountered in daily life), 2) enables introduction of repeated perturbations while eliminating the subject’s ability to perceive the obstacle’s entry and thus anticipate the events, 3) provides controllable timing of the perturbation, and 4) enables calculation of joint-level kinematics and kinetics before, during, and after the perturbation. The proposed system is based on and improves upon the system presented by [18–21]. However, the one described here precludes subjects from perceiving the deployment of the obstacle, is able to time the perturbation to within 2.5% of the stride cycle, and enables straightforward computation of joint kinetics using conventional inverse dynamics techniques. This paper describes the design of the system and employs it to collect kinematic and kinetic data on seven healthy subjects in order to confirm that the four aforementioned objectives are met. Apparatus design files and targeting algorithm scripts are included in the Additional files 2 and 3, respectively, such that other researchers can reproduce the experimental system for the study of stumble recovery.

## Methods

### Stumble perturbation system

**Fig. 1:**
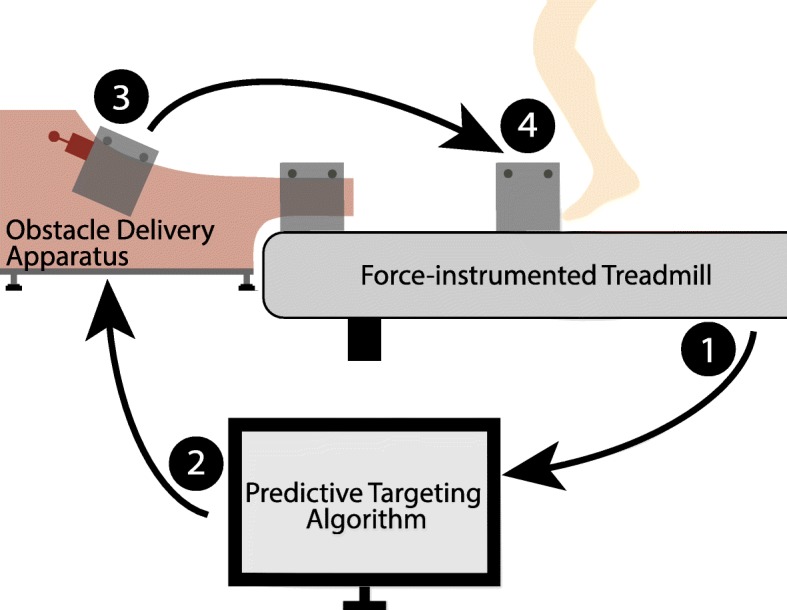
Schematic of the stumble perturbation system. The subject walks on the instrumented treadmill. Ground reaction forces and moments are collected (1) and used to calculate the center of pressure under the foot, which is then used to detect gait events. These gait events are used to calculate the time at which the obstacle should be released using the predictive targeting algorithm (2). At this time the electromagnet turns off (3) and releases the obstacle onto the treadmill such that a perturbation is introduced (4) at the desired percent of swing phase

The stumble perturbation system consists of (i) an obstacle delivery apparatus that inconspicuously releases an obstacle onto a split-belt, force-instrumented treadmill, and (ii) a predictive targeting algorithm which controls the timing of the perturbation. The major components of the system are illustrated in Fig. 1.

#### Obstacle delivery apparatus design

As with the system described in [18–21], the essential mechanism of inducing a stumble is based on introducing a weighted obstacle onto a treadmill belt. The obstacle used in this study was a 16 kg (35 lb) block of steel, chosen to ensure minimal movement relative to the treadmill belt during a stumble compared to previous designs which used a 2.2 kg (5 lb) obstacle [18–21]. The obstacle measures 20 cm wide, 12.5 cm long, and 7.5 cm high (8.125” x 5” x 3”). Firm foam padding of 1.25 cm in thickness (0.5”) is adhered to the front and bottom of the obstacle to protect the subject’s toes and the treadmill belt, respectively. Note that while this specific weight and shape were used for this study, an obstacle of any given weight and various shapes could be used in its place depending on the objective of the experiment. The system’s functionality is independent of the obstacle’s weight and shape, within certain bounds of size and form-factor.

Additionally, deployment of the obstacle onto the belt without perception necessitates that the obstacle be transferred to the belt with minimal impulse, which requires that the obstacle be deployed with near-zero vertical velocity, and horizontal velocity that approximates the treadmill belt speed. Any substantial variation from these velocities will result in a noticeable force impulse on the treadmill due to the change in obstacle momentum, which will be perceptible to the user, as was the case in previous designs [19, 20] and evident in the authors’ pilot testing.

**Fig. 2:**
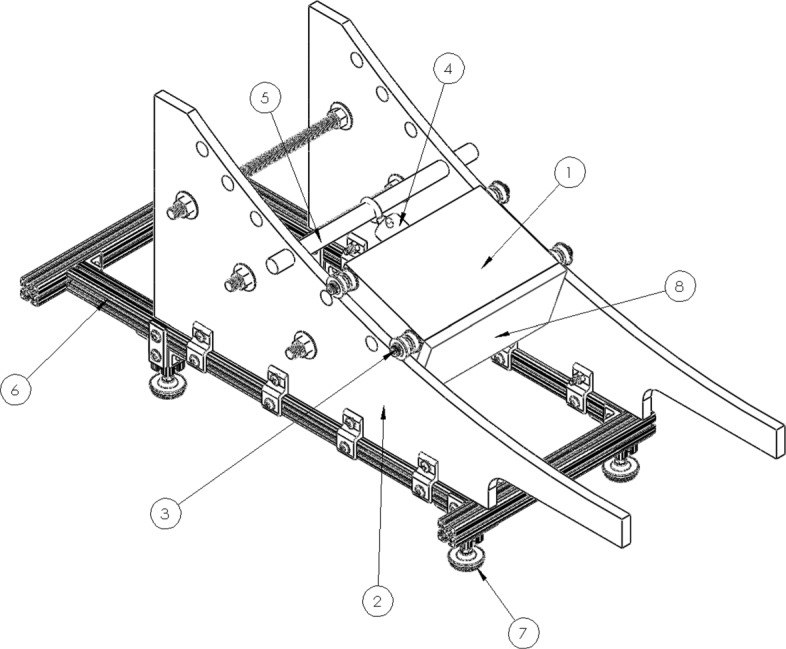
Obstacle delivery apparatus. A steel block (1) rests on an acrylic track (2) via flanged bearing stacks (3). The block is held in place by an electromagnet (4), whose position is determined by the height of the metal rod (5). The track is mounted to an aluminum frame (6) with adjustable, vibration-damping feet (7). Foam (8) is adhered to the front and bottom of the block to protect the subject’s toes and reduce the impulsive loading on the treadmill, respectively

In order to deploy the 16 kg obstacle with minimal impulse, a ramp-based obstacle delivery apparatus (Fig. 2) was designed to deploy the obstacle onto the treadmill belt, as illustrated in Fig. 1, at near-zero vertical velocity and at a prescribed, adjustable horizontal velocity in order to match a given belt speed. The ramp consists of an acrylic track attached to an aluminum frame with adjustable, vibration-damping feet. The obstacle is held at a given point along the ramp via an electromagnet, which is held by a rod located by a pair of holes in the ramp (Fig. 2). When released via computer control (discussed subsequently), the obstacle rolls down the ramped track on a set of flanged roller bearings mounted on shoulder bolts threaded into each corner of the obstacle (Fig. 2) and then onto the front of the treadmill belt (Fig. 1). Note that a large, padded bin was used to catch the obstacle on the posterior end of the treadmill. The initial height of the center of mass of the obstacle determines the horizontal velocity at exit, and thus the ramp includes multiple starting points for the obstacle (i.e., multiple initial heights) in order to approximate a range of treadmill belt speeds. The starting height can additionally be fine-tuned via a threaded interface between the electromagnet and the rod to more precisely match a given belt speed. While any curve that is tangent to the treadmill belt at the exit could be employed, in order to simplify and improve the timing algorithm, a tautochrone curve [32] was implemented, which has been shown to have two desired features for this application. For a mass without friction in a constant gravitational field, a tautochrone curve: 1) provides the fastest path between two points, and 2) provides a constant time of travel, regardless of the starting point. These features respectively: 1) minimize the delay between obstacle release and the perturbation, which reduces associated predictive error, and 2) enable multiple belt speeds while considering only a single, fixed ramp travel time in the algorithm.

#### Predictive targeting algorithm

**Fig. 3:**
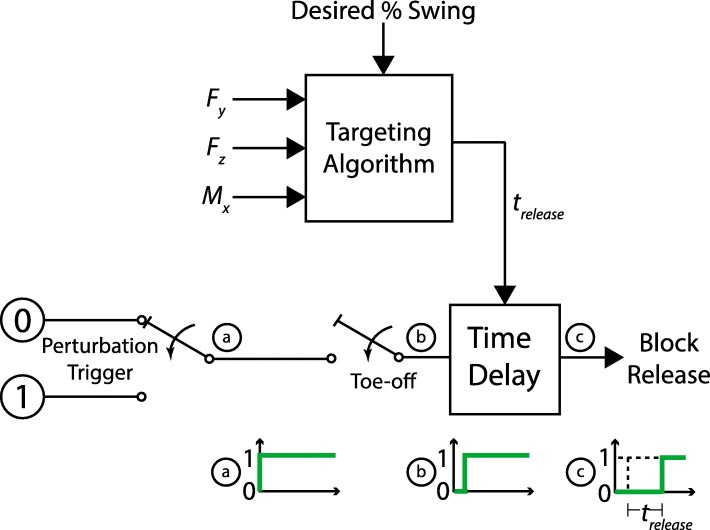
Predictive targeting algorithm control flow diagram. The targeting algorithm receives the desired percent swing input from the experimenter and the F_y_,F_z_, and M_x_ signals from the instrumented treadmill. Once the experimenter triggers a perturbation (a), the system waits until the next toe-off event (b), then passes to the time delay block where the time delay, *t*_*release*_, is received from the Targeting Algorithm. The system then releases the obstacle following the time delay (c) which results in a targeted perturbation during the subject’s swing phase. The unit step plots indicate the obstacle release signal at each point in the flow diagram: (a) indicates the immediate switch to high at the time of the trigger, (b) indicates the delay of the switch due to the time between the trigger and the subsequent toe-off, and (c) represents the added algorithmically-computed time delay before the obstacle is released. Note that *t*_*release*_ is calculated in Eqs. (1) – (10)

**Fig. 4:**
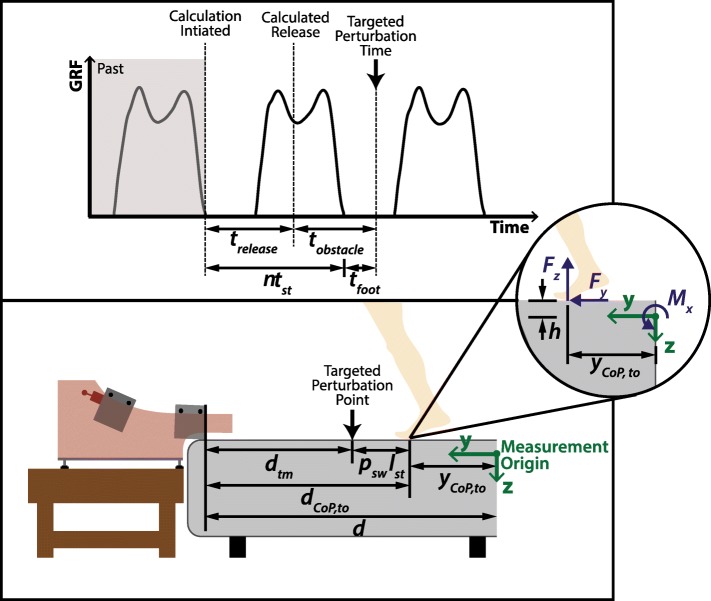
Schematic depicting variables used in the predictive targeting algorithm. The algorithm calculates the release time (*t*_*release*_) such that the obstacle contacts the subject’s foot at the desired time in swing phase. The time domain variables (top) and position domain variables (bottom) depicted here are used in Eqs. (1) – (10)

A predictive targeting algorithm was developed so the system could elicit precisely timed perturbations during a given stride, in order to: 1) reduce the proportion of mistrials (e.g., in [7, 19], where 23% and 39% of attempts failed to elicit a stumble, respectively), and 2) enable a more precise study of the variation in response mechanics as a function of when the perturbation occurs during swing phase. The targeting algorithm assumes the use of a lateral split-belt, force-instrumented treadmill. The control flow information is illustrated in Fig. 3. Note that both a left and right obstacle delivery apparatus were used for the human subject experiment described here, but each is independent (i.e., only requires kinetic signals from the side to be perturbed) and therefore the algorithm is described in the context of a single obstacle delivery apparatus. The predictive targeting algorithm is initialized with a desired percent swing at which the perturbation should occur. At the next toe-off event after the obstacle release is triggered by the experimenter, the algorithm calculates a time delay (*t*_*release*_) such that the perturbation will occur at the desired percent of swing phase. The desired percent of swing phase corresponds to a point in space and time after toe-off, hereafter referred to as the targeted perturbation point and the targeted perturbation time, respectively. The algorithm requires real-time measurement of the two sagittal plane forces (vertical and anterior-posterior (AP) ground reaction forces (GRF)) and the one sagittal plane moment (mediolateral ground reaction moment (GRM)) from the instrumented treadmill. These kinetic signals are used to calculate the AP center of pressure (CoP) which is then used to detect gait events. The detected gait events are used to determine the timing of the release of the obstacle to achieve a perturbation at the desired percent of swing phase. The specific algorithm by which the appropriate time delay is calculated is described below. The key measurements and variables used in the algorithm are defined in Fig. 4. For the implementation described here, the force and moment signals were sampled at 1 kHz and filtered with a 1st order low-pass filter with a cut-off frequency of 30.5 Hz. Additionally, an experimentally determined 90 N threshold was used on the vertical GRF to reduce noise in the CoP signal near heel-strike and toe-off. Below this threshold the CoP signal was zeroed. Also note, as indicated in Fig. 4, the sagittal plane forces and moment are measured by the instrumented treadmill with respect to a coordinate frame, which is provided by the treadmill manufacturer (Bertec, Columbus, USA).

The targeting algorithm assumes a uniform periodic motion (i.e., treadmill velocity is constant and the subject’s position on the treadmill does not change substantially between time of release and time of perturbation). The time delay *t*_*release*_ signifies when the obstacle should be released by the electromagnet in order to perturb the subject at a desired percent of swing phase. The time delay *t*_*release*_ is calculated at a specified toe-off as a function of several component times, illustrated in Fig. 4, as:

1 $$\begin{array}{@{}rcl@{}} t_{release} = {nt}_{st} + t_{foot} - t_{obstacle} \end{array} $$

where *t*_*st*_ is the average stride time, *t*_*foot*_ is the time required for the foot to travel from toe-off position to the targeted perturbation point, *t*_*obstacle*_ is the time required for the obstacle to travel from its initial position on the ramp to the targeted perturbation point, and *n* is the smallest integer that makes *t*_*release*_≥0. The component times in (1) are computed as follows. The time *t*_*obstacle*_ is defined by:

2 $$\begin{array}{@{}rcl@{}} t_{obstacle} = t_{tm} + t_{ramp} \end{array} $$

where *t*_*ramp*_ is the time required for the obstacle to travel down the ramp to its point of entry on the treadmill belt and *t*_*tm*_ is the time required for the obstacle to travel on the treadmill belt from its point of entry on the treadmill to the targeted perturbation point. The time *t*_*ramp*_ is a constant due to the nature of the tautochrone curve and thus is independent of the starting position of the obstacle on the ramp. Although this time can be estimated analytically, it was determined experimentally to account for frictional effects. The time *t*_*tm*_ is given by:

3 $$\begin{array}{@{}rcl@{}} t_{tm} = \frac{d_{tm}}{v_{tm}} \end{array} $$

where *v*_*tm*_ is the treadmill belt velocity, assumed to be constant, and *d*_*tm*_ is the distance the obstacle must travel on the treadmill to the targeted perturbation point, which is calculated as:

4 $$\begin{array}{@{}rcl@{}} d_{tm} = d_{CoP,to} - p_{sw}l_{st} \end{array} $$

where *d*_*CoP,to*_ is the computed distance from the obstacle’s point of entry on the treadmill to the CoP at the toe-off event, *p*_*sw*_ is the targeted percent of swing phase (converted to a decimal) which is provided as an experimenter input into the algorithm, and *l*_*st*_ is the computed average stride length (see Fig. 4). In this equation, the computed distance *d*_*CoP,to*_ is given by:

5 $$\begin{array}{@{}rcl@{}} d_{CoP,to} = d - y_{CoP,to} \end{array} $$

where *d* is the AP positional offset between the obstacle’s point of entry on the treadmill and the force plate origin (Fig. 4), and *y*_*CoP,to*_ is the distance from the AP CoP of the ipsilateral foot at toe-off to the force plate origin, which is given by calculating *y*_*CoP*_ at the time of toe-off using:

6 $$\begin{array}{@{}rcl@{}} y_{CoP} = \frac{F_{y}h+M_{x}}{F_{z}} \end{array} $$

where *h* is the vertical positional offset between the belt surface and force plate origin, *F*_*y*_ is the AP GRF, *F*_*z*_ is the vertical GRF, and *M*_*x*_ is the mediolateral GRM (Fig. 4).

The stride length in (4) is computed as a moving average of the previous 10 stride lengths, each of which is calculated as the difference of the AP CoP at heel-strike and the prior toe-off:

7 $$\begin{array}{@{}rcl@{}} l_{st} = y_{CoP,hs}(i) - y_{CoP,to}(i-1) \end{array} $$

where *i* is the stride index, and *y*_*cop,hs*_ and *y*_*CoP,to*_ are the distances from the AP CoP signal to the force plate origin at heel-strike and toe-off, respectively. The heel-strike event is detected as an increase in the vertical GRF beyond the 90 N threshold, and heel-strike position is computed as the average of the first 10 non-zero samples of the AP CoP signal after the heel-strike event. The toe-off event is detected as a decrease in the vertical GRF below the threshold, and toe-off position is computed as the last 10 non-zero samples of the AP CoP signal prior to toe-off. Both of these CoP values are calculated using (6) at the time of their respective events. Stride time in (1) is the measured time between each successive ipsilateral heel-strike:

8 $$\begin{array}{@{}rcl@{}} t_{st} = t_{hs}(i) - t_{hs}(i-1) \end{array} $$

which is computed as a moving average of the past 10 stride times. Heel-strike time (*t*_*hs*_) is the time of the heel-strike event, which is detected at the first non-zero sample of the AP CoP signal after swing phase.

The time *t*_*foot*_, which is the time within the periodic cycle required for the foot to advance from the toe-off position to the targeted perturbation point, is given by:

9 $$\begin{array}{@{}rcl@{}} t_{foot} = p_{sw}t_{sw} \end{array} $$

where *t*_*sw*_ is the moving average of the previous 10 swing times, each of which is calculated as the time difference between heel-strike and the prior toe-off:

10 $$\begin{array}{@{}rcl@{}} t_{sw} = t_{hs}(i) - t_{to}(i-1) \end{array} $$

where toe-off time (*t*_*to*_) is detected at the time of the last non-zero sample of the AP CoP signal during stance phase. Note that the calculation of swing time (*t*_*sw*_) may be affected by the threshold set on the force signals, thus artificially increasing the value. In this implementation an experimentally determined scaling factor that was inversely proportional to the subject’s average stride length was used to account for this effect of thresholding the GRF. As illustrated in Fig. 3, the time delay value calculated in (1) is computed at the toe-off event, using (2)-(10), to enable the experimenter to specifically target a perturbation at a desired percent of the swing phase. The computer-aided design (CAD) files for the obstacle delivery apparatus and scripts implementing the targeting algorithm are included in the Additional files 2 and 3, respectively, of this paper. The following section provides a validation of the experimental setup and algorithm.

### Experimental validation

A 7-subject study of stumble recovery responses in healthy subjects was conducted in order to validate the efficacy of the stumble perturbation system. The protocol for this study and data analysis methods are outlined respectively in the subsections below.

#### Experimental protocol & data collection

Seven subjects participated, three females and four males (age: 23.6 yrs, height: 1.8 m, mass: 81.3 kg). All experimental protocols were approved by the Vanderbilt Institutional Review Board, and all subjects gave their written informed consent. Subjects walked on the treadmill at 1.1 m/s [19]. The handrails were removed so they could not be used as a recovery aid; however, a full-body harness with slackened safety rope was worn to prevent a true fall. To prevent subjects from hearing or seeing the obstacle being deployed, each subject listened to white noise via earbuds, wore noise-canceling headphones, and wore dribble goggles that occluded the inferior visual field. Each subject watched on-screen visual feedback to ensure a centered position on the treadmill and avoid crossing over to the contralateral force plate. As a distraction technique, subjects were instructed to count backwards aloud from an arbitrary number by intervals of seven [33] (i.e., perform Serial Sevens). Subjects were given several minutes to walk on the treadmill prior to testing in order to acclimate to the setup.

Various data were recorded during each trial, including GRF data, which were recorded under each foot at a sampling rate of 2 kHz via a lateral split-belt, force-instrumented treadmill (Bertec, Columbus, USA). Full-body kinematic data were collected via infrared motion capture at a sampling rate of 200 Hz, which included feet, shanks, thighs, pelvis, torso, upper arms, and forearms (Vicon, Oxford, GBR). The experimental protocol consisted of two sub-experiments. First, a perception experiment (hereafter referred to as Perception Trials) was performed to determine the extent to which subjects could perceive the deployment of the obstacles due to the potential introduction of vibrations to the treadmill, as this perception would induce an anticipatory response. Second, a perturbation experiment (hereafter referred to as Perturbation Trials) was performed to assess the timing accuracy of perturbations and quantify the kinematics and kinetics of the stumble recovery responses. In the Perception Trials, for each of the two belts, an obstacle delivery apparatus was aligned laterally on the treadmill belt to ensure that when the obstacle was released it would not contact the subject’s foot (i.e., it would pass lateral to the foot path). Subjects walked for approximately 15 min while the obstacles were released 6 times per belt at approximately 20, 30, 40, 50, 60, and 70% of swing phase. The subjects were asked to raise their hand on the respective side if or when they perceived the obstacle entering the treadmill.

Following the Perception Trials, each obstacle delivery apparatus was repositioned such that the obstacles, when released, would be in the line of progression of the subject’s foot. During the Perturbation Trials, each subject was perturbed 14 times per lower limb, targeted from 10% to 75% of swing phase in 5% increments. The order of perturbations was randomized in terms of targeted percent swing, the number of strides prior to the perturbation (between 25 and 120) and the side perturbed (i.e., left vs. right). A video of representative stumbles from these trials is provided in the Additional file 1.

Note that prior to each trial the subjects were not informed when or on which side the obstacle would be released, and as such they did not know when or where to expect the perturbation. The instructions given to the subjects prior to the Perturbation Trials were as follows: 1) Watch the visual feedback on screen to ensure a centered position on the treadmill, 2) perform serial sevens out loud, and 3) when the perturbation occurs, try to recover and return to steady state walking. They were also informed that in the event of a fall (i.e., in which they were caught by the overhead harness), the treadmill would be stopped.

Additionally, 60 s of unperturbed walking data were collected before and after the set of 28 perturbations, and the Perception Trials were repeated after the Perturbation Trials to ensure subjects did not acclimate to the system.

#### Data processing

GRF and motion capture data were filtered with a zero-phase, 3rd order, low-pass Butterworth filter with a cut-off frequency of 15 and 6 Hz, respectively. Next, inverse dynamics were computed using Visual3D (C-Motion, Germantown, USA) to estimate joint-level kinematics and kinetics for each trial. Additionally, the kinetic signal profile of the obstacle (i.e., the GRF and GRM profiles due to the obstacle as it travels across the treadmill from entry to exit) was obtained prior to the testing session (i.e., without a subject on the treadmill) and subsequently subtracted from the kinetic signals recorded during the Perturbation Trials. This removed the obstacle’s contribution to the measured kinetic data.

Prior to analysis, unperturbed walking data were parsed by heel-strike into strides and normalized to 100% of the stride cycle. Perturbed strides were normalized such that the toe-off event matched that of the unperturbed walking strides (i.e., perturbed strides were normalized based strictly on the stance phase, which is devoid of the perturbation).

The percent swing at which the stumble actually occurred was calculated as:

11 $$\begin{array}{@{}rcl@{}} P_{sw} = \frac{t_{pto}}{t_{sw,avg}} \end{array} $$

where *t*_*pto*_ is the time the perturbation occurred relative to the preceding toe-off event, and *t*_*sw,avg*_ is the average swing time of 25 strides prior to the perturbation. The perturbation event was determined as the instant at which the foot contacted the obstacle, which was identified via a transient peak in the AP GRF measured by the treadmill. The actual percentage of swing phase of the perturbation, *P*_*sw*_, was then compared to the targeted percentage of swing phase of the perturbation, *p*_*sw*_, to assess the accuracy of the system.

The responses of the subjects to each perturbation were divided into three stumble recovery strategies that have been defined in previous works: the elevating strategy [5, 15, 20, 24], lowering strategy [5, 20, 24], and delayed lowering strategy [20, 24]. The recovery strategy was determined by the trajectory of the perturbed foot after contact with the obstacle. For the elevating strategy, the perturbed foot lifts up and over the obstacle, landing anterior to the obstacle. For both the lowering and delayed lowering strategies, the perturbed foot lowers posterior to the obstacle. During the delayed lowering strategy, the perturbed foot lifts slightly before lowering posterior to the obstacle, while in the lowering strategy the perturbed foot shows no upward movement before lowering.

To demonstrate that the system enables calculation of joint-level kinematics and kinetics, hip, knee, and ankle angle and moment trajectories over the recovery period for each strategy were computed and shown for a single subject in Figs. 6 and 9. To briefly summarize group-level results, peak GRFs, trunk deflection and joint flexion angles, and joint flexion and extension moments were also computed and the inter-subject means of these metrics for each recovery strategy were determined.

**Fig. 5:**
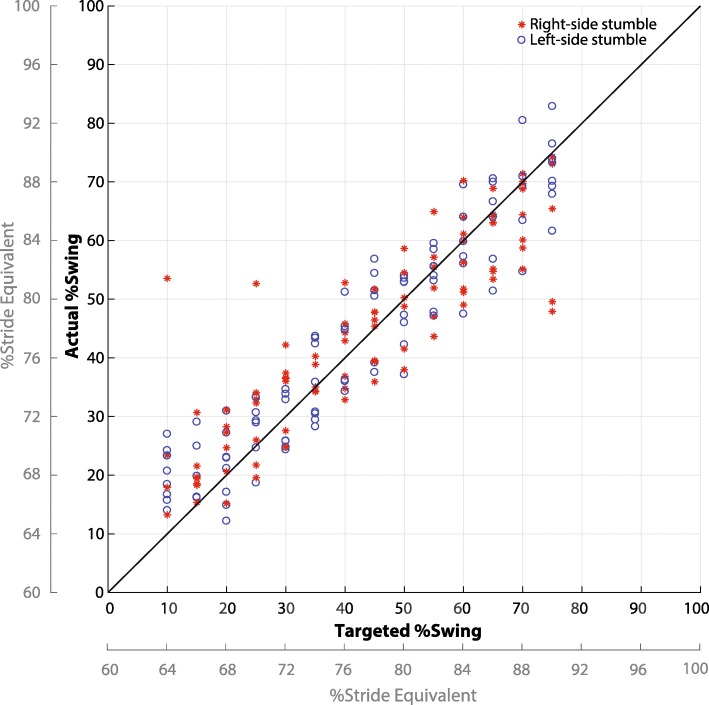
Targeted percent swing of perturbation versus the actual percent swing. Targeted percent swing is the input of the predictive targeting algorithm. Data are shown for 190 stumbles (28 trips per 7 subjects, excluding 6 mistrials). The mean absolute error of the system was 6.2% swing (2.5% stride), which corresponds to approximately 25 ms. An identity line is included to better visualize the system’s accuracy. The stride equivalent axis assumes swing phase makes up 40% of the stride cycle

**Fig. 6:**
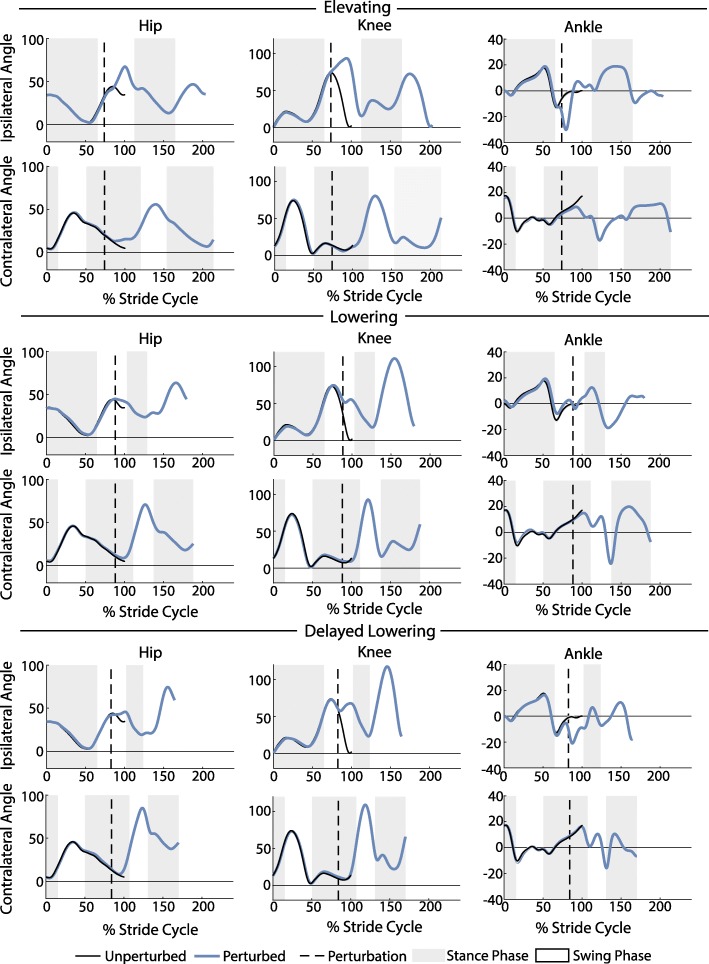
Kinematic trajectories of the hip, knee, and ankle. Depicted are the kinematic trajectories of the ipsilateral and contralateral limbs during an elevating, lowering, and delayed lowering strategy from a single subject. The trajectories were normalized to the toe-off of the unperturbed stride and extended accordingly. The unperturbed stride shown is the average of 25 strides prior to the perturbation. For the hip and knee, positive angles indicate joint flexion and negative angles indicate joint extension. For the ankle, positive angles indicate dorsiflexion and negative angles indicate plantarflexion. Angles are reported in degrees

These summary metrics served to 1) provide values with which to compare to previous overground studies as further validation that the system is able to provide realistic perturbations leading to authentic stumble recovery responses, and 2) present initial findings to show trends in magnitude and range of responses across different recovery strategies. Wilcoxon rank-sum tests (*α*=0.05) with a Holm-Bonferroni correction were used to determine statistical significance of these summary metrics for each recovery strategy compared to the unperturbed control values.

## Results

### Subject perception

Of the 168 obstacle deployments that occurred during the Perception Trials (12 pre-test and 12 post-test trials for 7 subjects), none were perceived by subjects.

### Targeting accuracy

The Perturbation Trials yielded 190 successful stumbles out of 196 attempted. Six trials were excluded due to the subject stepping onto the obstacle. Therefore, only 3% (6 of 196) of the trials were deemed mistrials. The authors note this failure rate is an order of magnitude lower than the overground experiments described in [7, 19].

Figure 5 shows the targeted versus actual swing percentage corresponding to the 190 successful stumble perturbations. The mean absolute error was 6.2% of swing phase (or 2.5% of the stride cycle, assuming that swing phase is approximately 40% of the total stride cycle). This corresponds to an average error of approximately 25 ms based on the average swing time (0.41 s ±0.027 s) from the 7 subjects.

### Kinematics

**Fig. 7:**
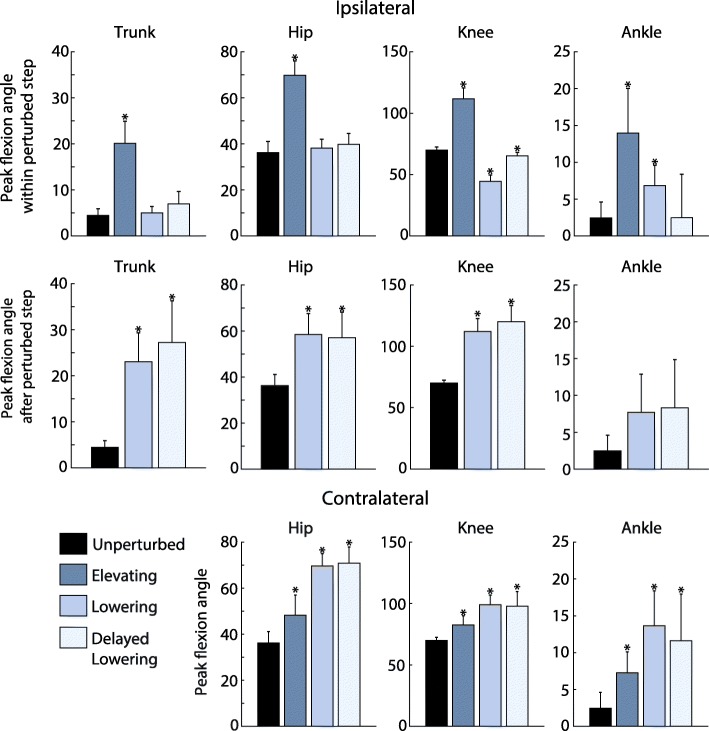
Peak joint and trunk deflection angles. Values are depicted for the elevating, lowering, and delayed lowering strategies on the ipsilateral and contralateral limbs. Each bar depicts the inter-subject (*N*=7) mean of the average peak angles for each strategy, with standard deviation displayed as error bars. For the ipsilateral (perturbed) limb, peak joint flexion angle or trunk deflection angle within the perturbed step (before the perturbed foot contacts the treadmill after perturbation) and after the perturbed step (in the step following the perturbation) are reported. For the contralateral limb, peak joint flexion angle in the step following the perturbation are reported. For the ankle, flexion refers to dorsiflexion. The unperturbed values shown are the inter-subject mean of the 25-stride average prior to the perturbation. Angles are reported in degrees. For each metric, values that are significantly different from that of unperturbed walking are marked with an asterisk

The observed recovery strategies and associated movements measured for the 7 subjects were qualitatively consistent with those previously reported for healthy individuals [5, 20, 24]. In total, 126 elevating strategies, 23 lowering strategies, and 39 delayed lowering strategies were observed. Two anomalous trials were excluded, one due to the subject scuffing his toe during the recovery, and the other due to the subject falling during the attempted recovery (i.e., caught by the safety harness). Sagittal plane kinematic trajectories for each of these strategies for a single subject are shown in Fig. 6, while inter-subject (*N*=7) mean peak joint angles and trunk deflection angle (i.e., the mean of each subject’s average peak angle for each strategy) are given in Fig. 7.

For the elevating strategy (Fig. 6 top row), the subject exhibited an increase in ipsilateral hip and knee flexion immediately following the perturbation as the leg cleared the obstacle. The ankle showed initial plantarflexion as the foot contacted the obstacle; however, this was quickly followed by dorsiflexion as the foot crossed over the obstacle. The contralateral limb kinematics did not deviate substantially from the unperturbed trajectories. The subject’s hip and knee flexion increased slightly in the step following the perturbation, along with a more substantial increase in ankle plantarflexion.

For the lowering strategy (Fig. 6 middle row), the subject exhibited ipsilateral hip extension after contact with the obstacle. In late swing phase, knee extension was exhibited as the foot lowered to the ground. There was slight ankle plantarflexion and initial knee flexion on the ipsilateral side as the foot impacted the obstacle. The ankle plantarflexion gave way to dorsiflexion before the foot hit the ground. In the following stride an increase in hip and knee flexion was seen while the ankle maintained slight dorsiflexion as the foot cleared the obstacle. The contralateral side displayed increased hip flexion and knee flexion during the following swing phase.

For the delayed lowering strategy (Fig. 6 bottom row), the subject’s response began with increased hip and knee flexion on the ipsilateral side, prior to switching to hip and knee extension as the foot was subsequently lowered to the ground. The ankle displayed initial plantarflexion as it contacted the obstacle before transitioning to dorsiflexion. In the next stride, an increase in hip and knee flexion and an increase in ankle dorsiflexion were observed as the foot cleared the obstacle. On the contralateral side, increased hip and knee flexion as well as ankle dorsiflexion were observed during the subsequent swing phase.

To complement the single subject results, Fig. 7 shows averaged results across all subjects in this study for various kinematic metrics associated with each strategy. During the perturbed step, the elevating strategy exhibited the highest deviation from unperturbed walking in peak trunk deflection angle (16^∘^ greater), ipsilateral peak hip flexion angle (34^∘^ greater), ipsilateral peak knee flexion angle (42^∘^ greater), and ipsilateral peak ankle dorsiflexion angle (12^∘^ greater). For the stride following the perturbation, the delayed lowering response exhibited the greatest deviation from unperturbed walking in peak trunk deflection angle (23^∘^ greater), ipsilateral peak knee flexion angle (50^∘^ greater), and ipsilateral peak ankle dorsiflexion angle (6^∘^ greater). On the contralateral side, the lowering strategy yielded the greatest deviation from unperturbed walking in peak knee flexion angle (28^∘^ greater) and peak ankle dorsiflexion angle (9^∘^ greater). Statistical significance for each metric compared to the unperturbed data is illustrated in Fig. 7.

**Fig. 8:**
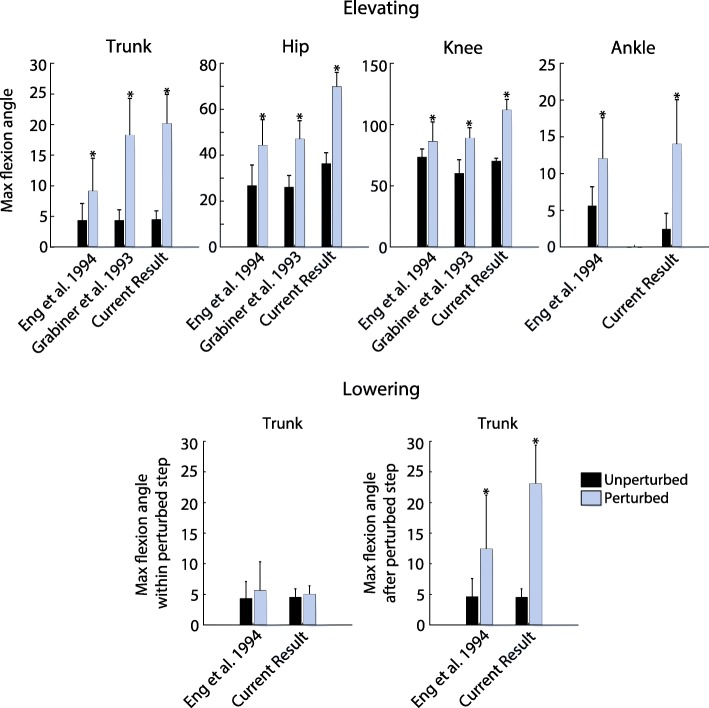
Peak joint angles for the elevating and lowering strategies. Results from this study as well as from previous works ([5, 15]) are reported. For this paper, the bars depict the inter-subject (*N*=7) mean of the average peak angles for each strategy, with inter-subject standard deviation displayed as error bars. For [5], the bars depict the total average peak angle for each strategy (*n*=25 stumbles for elevating, *n*=17 stumbles for lowering, *n*=25 for unperturbed control), with standard deviation displayed as error bars. For [15], the bars depict total average peak angles for the elevating strategy (*n*=42 stumbles), with standard deviation displayed as error bars. For the elevating strategy, peak flexion angle is defined as the peak joint angles of the ipsilateral limb and peak trunk deflection angle during the swing phase of the recovery. For the lowering strategy, peak trunk deflection angle within perturbed step (before the perturbed foot contacts the treadmill after perturbation) and after perturbed step (in the step following the perturbation) are reported. For the ankle, flexion refers to dorsiflexion. The unperturbed values shown for the current result are the inter-subject mean of the 25-stride average prior to the perturbation. Angles are reported in degrees. Asterisks above bars indicate that the value is significantly different from the same metric for unperturbed walking

Figure 8 compares summary metrics across all subjects in this study to the same metrics reported in previous overground studies, specifically those reported in [5, 15]. For the elevating strategy, the peak trunk deflection, hip flexion, knee flexion, and ankle dorsiflexion angles are qualitatively comparable to previous studies, although slightly larger. All metrics reported in both this paper and [5, 15] are significantly different from their respective unperturbed metrics for the elevating strategy. For the lowering strategy, trunk deflection angle during the perturbed stride was again qualitatively comparable to [5] (both this paper and [5] found no significant difference between this metric and that of the unperturbed stride). In the stride following the perturbation, trunk deflection angle is significantly different from that of the unperturbed stride for this paper and [5], though this paper reports slightly higher trunk deflection angle during the perturbed stride. Statistical significance for each metric compared to the unperturbed data for this paper and for [5, 15] is illustrated in Fig. 8.

**Fig. 9:**
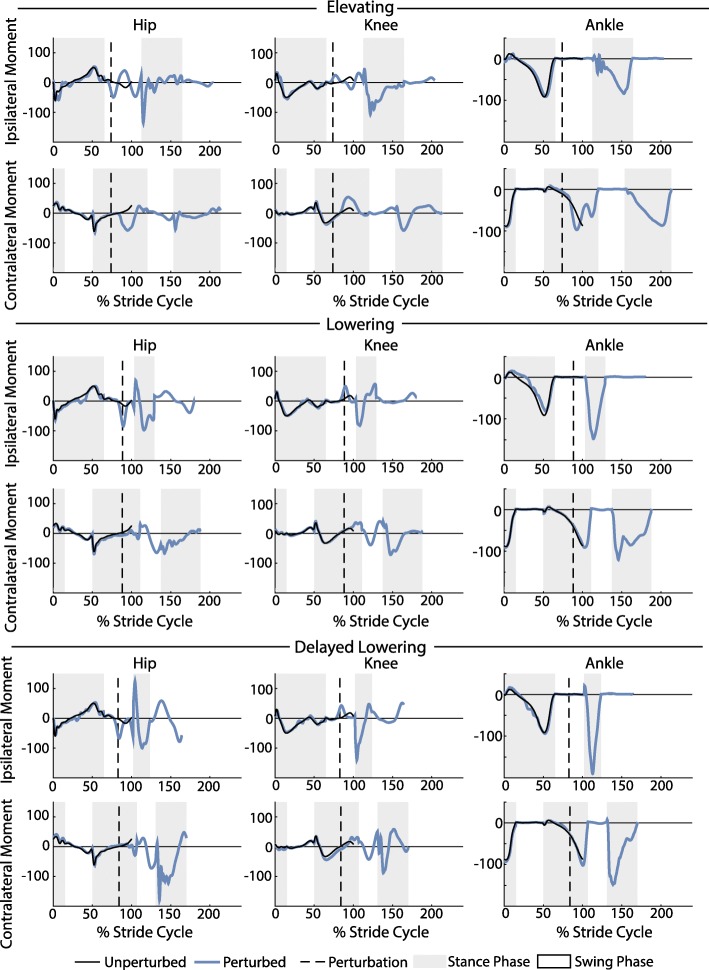
Kinetic trajectories of the hip, knee, and ankle. Depicted are the kinetic trajectories of the ipsilateral and contralateral limbs during an elevating, lowering, and delayed lowering strategy from a single subject. The trajectories were normalized to the toe-off of the normal stride and extended accordingly. The unperturbed stride shown is the average of 25 strides prior to the perturbation. For the hip and knee, positive moments indicate flexion moments and negative moments indicate extension moments. For the ankle, positive moments indicate dorsiflexion moments and negative moments indicate plantarflexion moments. Moments are reported in Newton-meters

### Kinetics

**Fig. 10:**
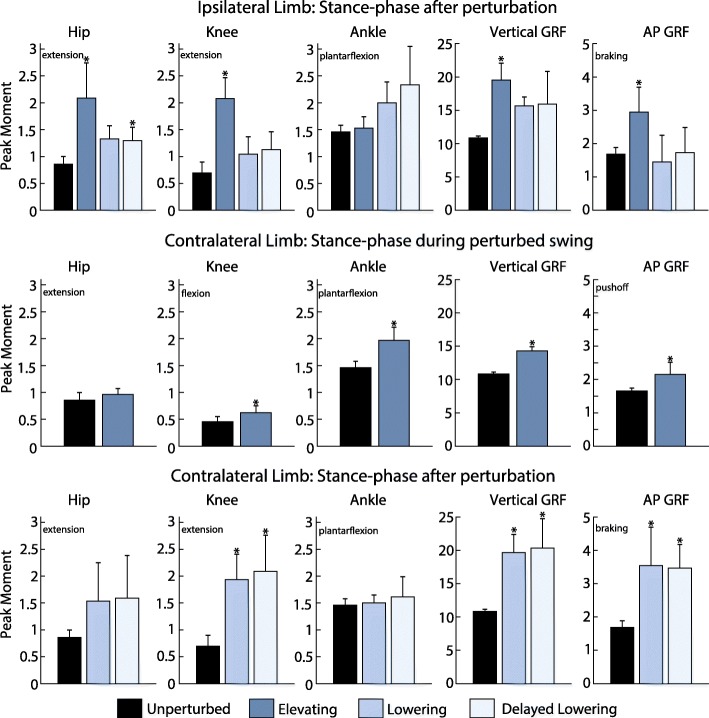
Peak joint moments and GRFs. Values are depicted for the elevating, lowering, and delayed lowering strategies on the ipsilateral and contralateral limbs. Each bar depicts the inter-subject mean of the average peak values for each strategy, with standard deviation displayed as error bars. The unperturbed values shown are the inter-subject mean of the 25-stride average prior to perturbation. Moments are reported as Newton-meters/kilogram, and forces are reported as Newtons/kilogram. Asterisks above bars indicate that the value is significantly different from the same metric for unperturbed walking

Joint-level kinetics were computed for each of the strategies for both the ipsilateral and contralateral limbs. Sagittal-plane joint moments for each strategy from the same subject are shown in Fig. 9. Inter-subject mean peak kinetic metrics across all subjects for each strategy as well as unperturbed walking are given in Fig. 10. Although the system enables the estimation of joint-level kinetics as intended, in several trials the subject crossed over onto the contralateral belt during recovery which compromises the inverse dynamics calculations, or the obstacle moved relative to the treadmill belt after impact with the swing foot (which would require an additional correction algorithm, not yet implemented, in order to properly estimate inverse dynamics). Because of these factors, trials in which either event occurred were removed from the kinetic analysis. As such, for the contralateral foot 126 (*N*=7) elevating strategies, 18 (*N*=7) lowering strategies, and 22 (*N*=6) delayed lowering strategies were included. For the ipsilateral foot 24 (*N*=7) elevating strategies, 2 (*N*=2) lowering strategies, and 16 (*N*=6) delayed lowering strategies were included.

For the elevating strategy (Fig. 9 top row), the subject exhibited a semi-periodic ipsilateral hip moment oscillating between extension and flexion during swing before producing a large extension torque upon contact with the ground. The ipsilateral knee displayed similar behavior as it oscillated between flexion and extension moments in swing before exerting a large extension moment upon heel-strike. These hip and knee moment behaviors are qualitatively consistent with the findings of [4]. During stance as the ipsilateral limb elevated over the obstacle, the contralateral limb experienced an extension moment at the hip and flexion moment at the knee, which is qualitatively consistent with the results of [13].

For the lowering and delayed lowering strategies (Fig. 9 middle and bottom row, respectively), the subject displayed very similar kinetic characteristics. After contact with the obstacle, the ipsilateral hip produced an extension moment before producing a flexion moment upon ground contact and then switching back to an extension moment in late stance. The ipsilateral knee demonstrated a flexion moment after contact with the obstacle before exhibiting an extension moment during early stance and switching to a flexion moment in late swing. The ipsilateral ankle demonstrated a large plantarflexion moment during stance after the perturbation. The contralateral limb initially exhibited a hip and knee flexion moment before entering swing phase and then exerted a large hip and knee extension moment on subsequent heel-strike.

Figure 10 shows averaged data across all subjects in this study for various kinetic metrics associated with each strategy. On the ipsilateral side, the elevating strategy exhibited the greatest deviation from unperturbed walking in peak hip extension moment (1.2 Nm/kg greater), peak knee extension moment (1.4 Nm/kg greater), peak vertical GRF (8.7 N/kg greater) and peak AP GRF (1.3 N/kg greater). The delayed lowering strategy exhibited the greatest increase in peak ankle plantarflexion moment (0.9 Nm/kg greater) on the ipsilateral side. As the perturbed limb elevates, the contralateral limb showed increases in peak hip extension moment, peak knee flexion moment, peak ankle plantarflexion moment, and peak GRFs compared to unperturbed walking. In the stance phase after perturbation, lowering and delayed lowering strategies exhibited increases in peak joint moments and GRFs on the contralateral side, compared to that of unperturbed walking. Significance for each metric compared to the unperturbed data is illustrated in Fig. 10.

## Discussion

The stumble perturbation system presented here provides a realistic obstacle perturbation to the swing foot, which both disrupts the foot’s trajectory and requires the person to clear a physical obstacle in the course of recovery to avoid falling. The system is capable of introducing repeated perturbations in a targeted manner without subjects being able to perceive or anticipate the perturbation. The system was able to elicit the three stumble recovery strategies previously described in literature, and kinematic and kinetic results obtained were qualitatively consistent with behaviors observed in previously published overground studies of stumble (Fig. 8). Further, the system enables precise, controllable timing of the perturbation to within 25 ms, or 2.5% of the stride cycle (Fig. 5). Finally, the system allows for the collection of joint-level kinematic and kinetic data for both limbs before, during, and after the perturbation (Figs. 6 and 9). The obstacle delivery apparatus design and predictive targeting algorithm are detailed in a manner to enable replication of the system for those wishing to study stumble recovery.

This system has eliminated the introduction of detectable vibrations to the treadmill, as evidenced by the Perception Trials, in which no subject indicated any perception of the obstacle out of 168 trials. Note that the Perception Trials were performed both before and after the Perturbation Trials, indicating that the subjects did not acclimate to the system. Avoiding perception of the obstacle prior to perturbation is imperative for the stumble perturbation system, as any anticipation of the impending perturbation will alter the reflexive nature of the stumble recovery response and compromise the authenticity of the response.

The apparatus itself has removed the issue of inducing vibrations to the treadmill, and thus the subject’s ability to feel the obstacle entering the treadmill. However, several other measures were taken to ensure that the subject was unaware of the obstacle’s entry (similar to previous works [19]). First, though efforts were made to reduce the audible noise from the obstacle rolling down the ramp (e.g., greased roller bearings), it is impossible to make such an event completely silent. Thus, white noise and noise-cancelling headphones were used. Second, the inferior visual field-blocking goggles were necessary to avoid subject temptation of looking down at the apparatus. Lastly, the Serial Sevens task was not necessary to avoid detection, but rather was chosen as a distraction task, since anticipation of stumble can alter the stumble response. The Serial Sevens task therefore was intended to distract and relax the subjects, in order to produce a more authentic stumble response.

The accuracy of the system in targeting specific times in swing phase helped to minimize mistrials and improve analytical resolution (Fig. 5). In this study, only 3% of trials were deemed mistrials (compared to [7, 19], in which 23% and 39% were mistrials, respectively). Minimizing mistrials is particularly important in the study of the stumble recovery response in populations with gait pathologies, for whom an excessive number of mistrials can lead to longer experiments, which can be physically taxing. Additionally, by ensuring the stumble perturbation system is accurate, a larger data set can be obtained without requiring as many sessions or as large of a subject pool.

The kinematic trends produced by the system (comparing unperturbed to perturbed strides) were qualitatively similar to those found in previous overground studies (Fig. 8), with the possible exception of peak hip flexion. The greater difference between unperturbed and perturbed peak hip angle is likely attributable to the fact that this study employed a heavier obstacle resulting in a greater impedance perturbation than from obstacles employed in the studies by [5, 15]. The similarity shown between the system depicted in this article and previous overground systems is notable as it demonstrates that the treadmill-based gait perturbation system provides kinematically and kinetically similar responses to overground stumble. Note that because the treadmill belt velocity was held constant for each trial, no acceleration was applied to the subject and as such the treadmill can be treated as an inertial reference frame, just as the ground is for overground walking.

The system allowed for the first time the calculation of ipsilateral and contralateral joint-level kinetics before, during, and after the stumble event. Time series data for a single subject and a brief set of kinetic summary metrics for all subjects are presented (Figs. 9 and 10). The purpose of this paper was simply to demonstrate the capability of the system. A more comprehensive analysis and interpretation of stumble recovery kinetics warrants further study but is beyond the scope and objective of this paper. One important consideration for future work is that the obstacle did not always remain stationary upon foot contact. Rather, in approximately 60% the recorded trips, the obstacle rotated about the vertical or mediolateral axis during the stumble. This behavior was similarly reported in other studies [19], and is representative of many actual stumble events (i.e., stumbling over a rock or heavy object that may shift on the ground). Movement of the obstacle, however, must be accounted for in the kinetic computations. A method for doing so could be developed and implemented using motion capture markers on the obstacle; however, it was not presented here, and therefore the ipsilateral kinetic data presented here (Fig. 10) corresponds to only the trials in which the obstacle did not move (70 of the 190 trials).

Beyond the limitation in kinetic analysis, other limitations of this system include the predictive targeting algorithm’s inability to respond to instantaneous changes in the subject’s gait immediately prior to or after the release of the obstacle and the assumption of constant foot velocity during swing phase. The predictive nature of the system (due to the travel time of the obstacle being notably longer than that of the subject’s foot) requires the system to take a moving average of several gait metrics which are used to determine the release time of the obstacle. Once the obstacle is released, the system cannot respond to deviations from the period motion, or belt location. Since the release occurs one to two strides prior to the perturbation, some deviations may occur, which are likely the limiting factor in determining targeting accuracy. Finally, the system’s assumption of constant foot velocity (in its calculation of the travel time of the subject’s foot) could also contribute to the slightly different levels of error in early and late swing (Fig. 5), since these regions are where this assumption is less robust.

The system’s efficacy in producing realistic, unanticipated, and controllable perturbations to the swing foot while allowing for the measurement and calculation of joint-level kinetics and kinematics lends itself to several prevalent research applications. First, its targeting capabilities could provide insight into several unknowns regarding the biomechanics of stumble recovery for healthy individuals, such as when and why healthy individuals choose specific recovery strategies. Second, this system can be used to study individuals who are particularly susceptible to perturbations of the swing foot (e.g., transfemoral prosthesis users) to study how their strategies differ from healthy individuals, and ultimately how to inform better interventions (e.g., prostheses) to recover from such perturbations. Lastly, as proposed and described by others, this system could be used for fall training purposes [34]; however, the efficacy of using this apparatus as a training device to potentially reduce the incidence or severity of community falls is unknown.

## Conclusion

The stumble perturbation system described in this paper was shown to be an effective means of inducing stumbles and evaluating recovery strategies during human walking. The system provided realistic obstacle perturbations and was also shown to prevent anticipation by the subjects due to the obstacle’s imperceptible entry onto the treadmill. The accurate targeting demonstrated by the system enables efficient and systematic data collection, thus reducing the required subject sample size or the amount of time spent collecting data on each subject. The ability to collect kinematic and kinetic data for both the ipsilateral and contralateral lower limbs allows for versatile use of the system across a diverse range of studies. Finally, this work provides the necessary obstacle delivery apparatus CAD files and predictive targeting algorithm scripts in Additional files 2 and 3, respectively, such that this system could be recreated and adopted by other researchers studying stumble recovery.

## Additional files

Additional file 1 A compilation video of a single subject recovering from a range of variously timed perturbations throughout swing phase. The subject is walking at 1.1 m/s. Elevating, lowering, and delayed lowering strategies are demonstrated. (ZIP 16,403 kb)

Additional file 2 CAD files detailing the design of the Obstacle Delivery Apparatus in full (ramp, block, and magnet). McMaster-Carr part numbers are included in the properties for applicable parts. (ZIP 37,588 kb)

Additional file 3 A script containing the essential functions to the operation of the Predictive Targeting Algorithm. Intended as a guide for similar systems. (C 31 kb)
